# Factors Affecting Recurrence of Idiopathic Granulomatous Mastitis: A Systematic Review

**DOI:** 10.1155/2023/9947797

**Published:** 2023-09-26

**Authors:** Asieh Sadat Fattahi, Ghasem Amini, Fatemeh Sajedi, Hassan Mehrad-Majd

**Affiliations:** ^1^Endoscopic and Minimally Invasive Research Center, Department of Surgery, Faculty of Medicine, Mashhad University of Medical Sciences, Mashhad, Iran; ^2^Department of Persian Medicine, School of Persian and Complementary Medicine, Mashhad University of Medical Sciences, Mashhad, Iran; ^3^Clinical Research Development Unit, Ghaem Hospital, Faculty of Medicine, Mashhad University of Medical Sciences, Mashhad, Iran

## Abstract

Idiopathic granulomatous mastitis is a rare and benign disease that primarily affects young women of reproductive age. Various factors have been suggested as possible causes, including pregnancy, breastfeeding, history of taking birth control pills, hyperprolactinemia, smoking, and history of trauma. Due to unknown etiology, opinions on its treatment have varied, resulting in differing recurrence rates and side effects. Therefore, conducting a comprehensive systematic review and meta-analysis can aid in understanding the causes and recurrence of the disease, thereby assisting in the selection of effective treatment and improving the quality of life. A systematic literature review was conducted using predefined search terms to identify eligible studies related to risk factors and recurrence up to June 2022 from electronic databases. Data were extracted and subjected to meta-analysis when applicable. A total of 71 studies with 4735 patients were included. The mean age of the patients was 34.98 years, and the average mass size was 4.64 cm. About 3749 of these patients (79.17%) were Caucasian. Patients who mentioned a history of pregnancy were 92.65% with 76.57%, 22.7%, and 19.7% having a history of breastfeeding, taking contraceptive pills, and high prolactin levels, respectively. Around 5.6% of patients had previous trauma. The overall recurrence rate was 17.18%, with recurrence rates for treatments as follows: surgery (22.5%), immunosuppressive treatment (14.7%), combined treatment (14.9%), antibiotic treatment (6.74%), and observation (9.4%). Only antibiotic and expectant treatments had significant differences in recurrence rates compared to other treatments (*p* value = 0.023). In conclusion, factors such as Caucasian race, pregnancy and breastfeeding history, and use of contraceptive hormone are commonly associated with the disease recurrence. Treatment should be tailored based on symptom severity and patient preference, with surgery or immunosuppressive options for recurrence.

## 1. Introduction

Idiopathic granulomatous mastitis (IGM) is a rare and benign inflammatory mammary disease with an unknown etiology [1]. Also known as granulomatous lobulitis or lobular granulomatous mastitis [2], it can be misdiagnosed as furuncle or cellulitis [3]. IGM primarily affects premenopausal women, typically between the ages 32 and 36 [4]. Various studies have identified factors that may contribute to the development of IGM, such as age, recent pregnancy [5, 6], duration of breastfeeding, history of contraceptive pills usage [6–9], smoking, and trauma [9, 10]. Hyperprolactinemia and diabetes have also been linked to the disease [5]. It is thought that milk stasis after pregnancy and lactation, combined with hyperprolactinemia, can lead to hypertrophic breast tissue and the development of IGM [1]. While IGM has been reported in all races [8], it appears to be more prevalent in certain regions, including the Mediterranean and Asian regions [9].

The disease's clinical manifestation is similar to a breast lump with inflammatory symptoms and abscess [11, 12] and is typically located in the breast's upper outer quadrant. Occasionally, it can be found in other quadrants, and in some cases, bilaterally [11]. The likelihood of both breasts being affected simultaneously is minimal [9], but the left breast is more commonly involved [2]. Unfortunately, this disease has no cure [3, 12]; however, in minor cases, it may resolve on its own without any treatment. For severe cases, medical or surgical intervention may be required [13].

Decisions regarding the management of this condition are dependent on the severity of the disease as well as the patient's preferences. Surgery still plays an integral role in its disease [3]. Despite receiving appropriate treatments, these masses may persist, recur, or sometimes lead to the development of fistulas, necessitating follow-up in affected patients [14, 15]. Moreover, the presence of erythema nodosum can further complicate the disease, making its treatment challenging [12].

In general, despite numerous studies in this field, a specific reference for the possible causes of this disease has not yet been defined. Although several risk factors have been proposed, there is no comprehensive summary that reviews the available evidence or a systematic review of studies. In addition, there is no consensus on a treatment method. Therefore, we conducted this comprehensive systematic review and meta-analysis to evaluate the risk factors for IGM and assess the likelihood of recurrence.

## 2. Methods

We conducted this review following the Preferred Reporting Items for Systematic Reviews and Meta-Analyses (PRISMA) guidelines to identify studies relevant to underlying factors and recurrence in patients with granulomatous mastitis.

**Figure 1:**
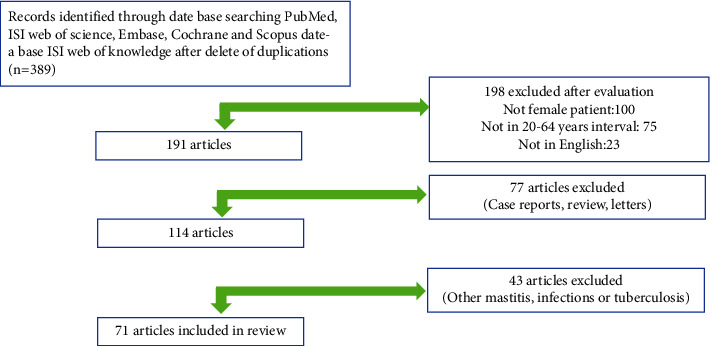
Flowchart of the study selection process for this review.

Two investigators performed a comprehensive electronic database search, including PubMed, Cochrane, Embase, and Google Scholar databases. The search period covered up to June 2022, and there was no time limit in the study selection. Our searches were conducted individually or in different combination using the following terms: “mastitis,” “granulomatous lobular mastitis,” “idiopathic granulomatous mastitis,” “recurrence,” “risk factors,” or etiology. We identified some additional studies from other sources and excluded studies with insufficient information. Only studies published in English were considered for this review. The selection process for the studies is summarized in Figure 1.

This study included published studies that satisfied the given criteria. Studies related to idiopathic granulomatous mastitis that were conducted with clinical trial methods, descriptive, cohort with or without control, and case control were included in this study. However, studies that described granulomatous mastitis in male or transgender patients were excluded. In addition, letters to the editor, review articles, and clinical reports with less than 4 patients were not included. Furthermore, studies that focused on other types of mastitis, articles that solely discussed radiological manifestations, articles without clinical or therapeutic explanations, and articles in languages other than English were also excluded. The following data were extracted for each study: name of the first author, country of origin of the study, year of publication, and the number of patients. The outcome measures that were extracted were the age of presentation, any association with autoimmune diseases, smoking habits, history of trauma, parity, lactating history, BMI, prolactin levels, clinical symptoms (i.e., presence of a mass, pain, or other symptoms), recurrence, and treatment (antibiotics, corticosteroids, or surgery).

In situations where quantitative data were appropriate for meta-analysis, the averages and ranges were calculated considering the analytic weight. The analytic weight is determined by the inverse proportionality to the variance of each observation. Typically, the observations represent averages, and the weights correspond to the number of elements contributing to each average. All statistical analyses were performed using SPSS 25 software. Finally, we interpreted the obtained results within the framework of the study's overarching criteria.

## 3. Results

Out of the 71 studies reviewed, a total of 4735 patients were included in this study (Table 1). Of these, 69 articles (4566 patients) reported the average age of patients at the time of symptom onset. The average age across these articles was 34.98 years. The incidence of the disease across different age ranges was not investigated. Racial distribution was reported in all 4735 patients, with 3749 (79.17%) being Caucasian, 543 (11.46%) Asian, and 350 (7.39%) Hispanic. There were also 38 Indian, 20 Moroccan, 14 African American, and 21 Jewish patients. Of the 55 publications that examined pregnancy history, 3251 out of 3554 reported on this aspect, accounting for 91.47% of the patients.

Out of the 71 articles, 44 of them mentioned the history of breastfeeding, while only 27 of them did not mention. Among the 3252 patients that were examined in those 44 articles, 2721 of them had a positive history of breastfeeding, indicating an 83.67% rate of breastfeeding. In addition, about 41 articles discussed the history of taking birth control pills, with a total of 3203 patients being examined. Of those patients, 730 had a history of taking contraceptive pills, accounting for 22.79% of patients. The smoking history of patients was examined in 38 studies, covering 2687 patients. Among these patients, 373 had a history of smoking, which is 13.88% of patients. Furthermore, 16 articles mentioned the history of trauma, manipulation, or surgery in the breasts, including a total of 628 patients. Among these patients, 36 had a positive history of the same, amounting to 5.7%. Prolactin tests were performed in 16 studies with a total of 502 patients being subjected to testing. Among them, 99 patients had high prolactin levels, accounting for 19.7% of patients (19.7%) (Table 2).

Out of the 71 included articles, 37 articles reported the granulomatous mastitis mass sizes for a total of 2151 patients. The average mass size for these patients was calculated to be 4.64 cm. In 10 studies, the body mass index (BMI) of the patients was discussed, with 4 studies reporting on the average BMI of 198 patients which was found to be 28.24. In addition, 4 articles examined the BMI in two age ranges—less than 25 years and more than 25 years—for a total of 146 patients. Among these patients, 37.67% had a BMI less than 25, while 62.33% had a BMI more than 25. Five articles studied the BMI in different ranges less than and greater than 30, with a total of 231 examined patients. Among these patients, 127 (55%) had a BMI less than 30 and 104 (45%) had a BMI greater than 30. A total of 60 articles examined recurrence, reporting on 4038 patients, with 694 cases experiencing recurrence, resulting in a calculated recurrence rate of 17.18% (Table 2). The variables were also studied based on the Caucasian race and other races, and there was no significant relationship with relapse (*p* = 0.171) (Table 3). The prevalence of different symptoms in patients (mass, pain, inflammatory symptoms, lymphadenopathy, skin symptoms, and systemic and infectious symptoms) in the Caucasian race and other races was compared. Except for which is significantly higher in other races than the Caucasian race (*p* value = 0.003), the prevalence of symptoms other than lymphadenopathy in the Caucasian race was not significantly different from other races (*p* > 0.05). However, lymphadenopathy was found to be significantly higher in other races than in the Caucasian race (*p* = 0.003).

To categorize the various treatments available for granulomatous mastitis, they were divided into five distinct groups. The first group consisted of surgical procedures, such as drainage, excision, lumpectomy, and others. The second group of treatment was immunosuppressive drug treatment including prednisolone, methotrexate, and so on. The third group included combined surgical and immunosuppressive treatments. The fourth group was dedicated to observation, while it focuses solely on antibiotic treatment. Many patients initially received antibiotic treatment before biopsy or any other treatment. Patients who did not undergo any other therapy, such as surgery or immunosuppressive drug therapy, were placed in the antibiotic group.

The overall recurrence rate among patients was found to be 17.18%. Among different treatment options, the recurrence rate of surgical treatment alone was 22.5%, the recurrence rate for immunosuppressive treatment was 14.7%, the recurrence rate for combined treatment was 14.9%, the recurrence rate for observation was 9.4%, and the recurrence rate for antibiotic treatment alone was calculated to be 6.74%.  Table 4 provides a detailed breakdown of these results. Only the recurrence rates for antibiotic and expectant treatment showed significant differences when compared to other treatments (*p* = 0.023). In addition, when comparing the recurrence rates of all treatments with those for surgical treatment alone, it was found that the recurrence rate for antibiotic and expectant treatments exhibited a significant difference compared to the recurrence rate for surgical treatment.  Table 4 provides further details on these comparisons.

## 4. Discussion

This study reviewed 71 articles and 4735 patients with granulomatous mastitis, a chronic inflammatory breast disease of unknown etiology. The incidence of this disease has increased in recent years (9 studies before 2010 compared to 62 studies after 2011), presenting a diagnostic and treatment challenge for clinicians. Our results supported that the disease is four times more prevalent in the Caucasian race, especially in the Mediterranean and Middle East regions, and commonly affects women in their reproductive age. Pregnancy (91.4%) and breastfeeding (83.6%) were the main underlying factors for the disease, followed by use of oral contraceptive pills (OCP) and high prolactin levels. Smoking, trauma, and weight gain had minimal association with the disease.

After considering the epidemiological and racial differences, a reanalysis was conducted on the underlying causes, symptoms, and recurrence rates between the Caucasian race and other races for IGM. The study found that there was no significant difference between the main causes, namely, pregnancy, breastfeeding, and hormone intake in two groups. Therefore, additional cohort and prospective studies are required to investigate other genetic or environmental factors such as nutrition and lifestyle, which might explain why the prevalence of IGM is four times higher in the Caucasian race. In terms of disease manifestation, mass disease purification was the most common symptom, followed by pain, inflammatory symptoms, lymphadenopathy, and skin involvement, respectively. A comparative analysis between Caucasian and other races exhibited similar clinical symptoms between the two groups, except for lymphadenopathy, which was more frequently observed in the non-Caucasians (*p* = 0.003). However, since only two studies mentioned this in the group of other races (43 patients out of 108 patients), and with the sample size being small, this outcome needs to be interpreted with caution. Regarding recurrence, an average of 17% of patients experienced it, in all the studies, indicating that current treatments are not associated with complete disease eradication and there could be a recurrence. Even though the recurrence rate is higher in the Caucasian group, it is not statistically significant, and it seems that the race or geographic distribution does not directly correlate with recurrence.

Various treatments have been mentioned for this disease, ranging from observation to systemic immunosuppressive treatment and surgery. Thus, we classified the treatments into several categories, including surgical treatment, immunosuppressive treatment, combined treatment, antibiotic treatment, and expectant treatment. As most patients received a course of empiric antibiotics before biopsy and diagnosis, we have classified studies in the antibiotic category, which only continued antibiotic treatment and did not receive any other systemic treatments or surgery.

Our results showed that surgery alone, immunosuppressive treatment alone, and combined treatment (surgical + immunosuppressive) did not have a statistically significant difference in recurrence rates. However, surgery without immunosuppressive treatment had a slightly higher recurrence rate, although the difference was not significant. Combined treatment may have treatment-related complications but did not statistically significantly reduce the recurrence rate. In line with our results, Li (review of 15 articles) [81] and Xiaojia (review of 21 articles) [82] mentioned that surgery was the best treatment for faster complete remission and adding steroid treatment did not make a difference in complete remission. They also reported that the rate of surgical recurrence was small and recommended medical treatment for patients concerned about scarring or future breastfeeding.

Furthermore, in our review, antibiotic treatment alone (without immunosuppression and surgery) and expectant treatment had a lower recurrence rate. However, the number of articles compared to other treatments in each category was small; 5 studies with 53 observed patients and 12 studies with 178 patients treated with antibiotics. Lei et al. [82] recommended systemic steroid treatment in the case of symptoms involving the whole breast, such as inflammation, skin involvement, and multiple fistulas. They also suggested this treatment in patients with limited symptoms at the beginning of treatment [81, 82]. Therefore, treatment should be chosen based on the patient's preference, severity of symptoms, and the physician's opinion while considering side effects related to each category such as scarring, deformity, and immunosuppressant drug treatment's side effects and longer treatment duration.

In conclusion, our review of IGM disease has identified that factors such as Caucasian race, pregnancy and breastfeeding history, and use of contraceptive hormone are commonly associated with the disease. Symptoms and manifestations do not appear to vary significantly across different races. Incidence rates appear to be increasing as more studies are carried out. With regard to treatment and recurrence, we suggest that a treatment regimen such as surgery or immunosuppressive treatment should be selected based on the severity of symptoms and patient's preference, taking into consideration the complications of each treatment line. Antibiotic and expectant treatments may be used initially and for minor symptoms.

## Figures and Tables

**Table 1:** Characteristics of studies included and outcome measure.

	Ref	Country	*N*	Mean age	Rec	f/*u* (m)	Conclusions	Study type	Study quality
1	Al Nazer, 2003 [16]	Saudi Arabia	11	35	3	—	Granulomatous mastitis is not unheard of and clinicians should keep it in their list of differential diagnosis of breast lumps	Case series	8
2	Bani-Hani et al., 2004 [17]	Jordan	24	34.3	4	31.2	A greater awareness of the rare entity of idiopathic granulomatous mastitis is mandatory to avoid unnecessary mastectomies	Cohort	7
3	Eric et al., 2004 [18]	China	9	45.7	4	18.7	It is important to differentiate IGM from carcinoma of the breast. Tissue biopsy remains the gold standard to confirm the diagnosis	Case series	7
4	Baslaim et al., 2007 [19]	Saudi Arabia	20	34	0	24	Management of IGM cases needs to be tailored according to the clinical presentation	Cohort	6
5	Uriel Katz et al., 2007 [20]	Israel	4	36.25	1	22	Clinicians should consider the possibility of idiopathic granulomatous mastitis in young women with inflammatory breast processes and negative findings on relevant biopsy, laboratory, and imaging studies	Case series	8
6	Al-Khaffaf et al., 2008 [21]	Saudi Arabia	18	36	3	—	IGM patients are younger, have given birth more recently, and are less likely to be Caucasian as compared with PDM patients	Cohort	8
7	Goldman et al. 2009 [22]	USA	7	32	—	—	When an idiopathic condition such as IGM is identified, a thorough clinical history and evaluation should be conducted to exclude known causes of granulomas	Case control	6
8	Nemenqani et al. 2009 [23]	Saudi Arabia	7	38	—	—	The definitive diagnosis can be established by a combination of the cytomorphologic features and microbiologic studies	Cohort	6
9	Kok and Telisinghe, 2010 [24]	Brunei	43	34	10	15	Complete surgical excision or incision and drainage of the lesion are the main treatment modalities	Cohort	7
10	Sakuraiet al., 2011 [25]	Japan	8	44.8	1	12	Steroid therapy was effective for the treatment of IGM	Cohort	6
11	Akbulut et al., 2011 [26]	Turkey	4	33.25	0	5.25	MTX in the present cases of IGM was effective, prevented complications, and limited corticosteroid side effects	Case series	7
12	Lacambra et al., 2011 [27]	China	33	37.85	0	—	Histologically, IGM is associated with more plasma cells	Cohort	6
13	Boufettal et al., 2012 [28]	Morocco	20	38.1	4	—	IGM is a rare entity. The treatment is medical alongside surgical excision	Cohort	6
14	Hugon-Rodin et al., 2012 [29]	France	14	33	13	61.5	Medical treatment with steroid reduces the duration and number of episodes. It also prevents the need for invasive breast surgery	Case series	8
15	Altintoprak et al., 2013 [13]	Turkey	26	37.5	4	38.4	This study was not able to support the eventual existence of an autoimmune basis of IGM.	Cohort	6
16	Neel et al., 2013 [30]	France	23	39	18	95.8	The value of immunomodulatory drugs such as colchicine or hydroxychloroquine deserves further investigation	Cohort	7
17	Omranipour et al., 2013 [31]	Iran	43	33.5	7	16	We hypothesized that a shorter lactation period may cause more milk stasis and extravasation and be contributory to IGLM	Cohort	6
18	Oran et al., 2013 [32]	Turkey	46	33	8	35.4	Treatment can be designated according to the extent and the severity of the disease	Cohort	7
19	Akahane et al., 2013 [33]	Japan	12	36	2	22	These results suggest that steroid treatment may be the first choice in treatment strategies for GLM	Cohort	6
20	Hur et al., 2013 [34]	South Korea	50	37.1	3	32	Surgery may play an important role when a lesion is determined to be mass forming or appears localized as an abscess pocket during breast examination or imaging study	Cohort	6
21	Cornejo-Juárez et al., 2014 [35]	Mexico	58	38	0	16.7	Biopsy is the gold standard for its diagnosis and should be taken in any patient even with a mild suspicion of cancer	Cohort	7
22	Kiyak et al., 2014 [36]	Turkey	24	38.4	1	38.4	Histopathologic confirmation is mandatory to ensure that a malignancy is not missed	Cohort	6
23	Salehi et al., 2014 [37]	Iran	59	32.48	34	12	This clinical trial demonstrated that pharmaceutical treatment has appropriate efficacy, in treatment and prevention of IGM relapse	RCT	3
24	Pandey et al., 2014 [38]	USA	49	35	9	9	Treatment with steroids is an effective breast-conserving option	Cohort	7
25	Mahlab-Guri et al., 2015 [39]	Israel	17	44.6	—	56.4	The recommended treatment modalities are observation alone or corticosteroids; surgery should be avoided	Cohort	8
26	Sheybani et al., 2015 [7]	Iran	22	32.82	3	11.91	Corticosteroids and MTX, with or without surgery, are the treatment of choice in these patients	Cohort	7
27	Atak et al., 2015 [40]	Turkey	40	39.07	14	24.85	Surgical excision still seems to be the best treatment method for IGM patients	Cohort	5
28	Bouton et al., 2015 [41]	USA	37	33	3	7.4	IGM is a self-limited benign condition that will resolve spontaneously without treatment	Cohort	6
29	Gopalakrishnan et al., 2015 [42]	India	38	32.85	1	24	The combination of limited surgical treatment and systemic prednisolone given for 6 months effectively controls the disease as well as prevents recurrence	Cohort	5
30	Mizrakl et al.i, 2015 [43]	Turkey	49	34.3	—	—	Systemic therapy with corticosteroids is an effective and appropriate treatment option for IGM. It can provide complete disease resolution and prevent recurrence	Cohort	6
31	Skandarajah and Marley, 2015 [44]	Australia	17	40	5	6	Exclusion of underlying systemic autoimmune conditions and judicious use of steroids and steroid-sparing agents such as methotrexate	Cohort	8
32	Yabanoğlu et al., 2015 [45]	Turkey	77	36.8	9	8.7	A wide surgical excision is the preferred approach for treating patients with IGM because of the low recurrence rate	Cohort	5
33	Aghajanzade et al., 2015 [14]	Iran	206	32	11	9–18	Corticosteroids are the first line of treatment with a good therapeutic response	Cohort	6
34	Ahmed and Maksoud, 2016 [46]	Egypt	13	35.53	2	24	Using therapeutic mammoplasty techniques in surgical management of IGM in moderate to large breasts seems justifiable with good results regarding recurrence and postoperative patients' satisfaction	Cohort	5
35	Elzahaby et al., 2016 [47]	Egypt	30	33	1	19	History of breast feeding together with early failure of complete nursing from a single breast is the most important risk factors for development of IGM in young women	Cohort	5
36	Helal et al., 2016 [48]	Egypt	65	38	5	—	This study supports the few recent studies that have detected GPB in IGM with cystic vacuoles	Cohort	4
37	Velidedeoglu et al., 2016 [9]	Turkey	10	38.4	1	21	Surgical management should be avoided unless all medical treatment options have been exhausted	Cohort	6
38	Calis and Karabeyoglu, 2017 [49]	Turkey	19	44	3	11	Patients who followed up by observation should be explained that IGM is a chronic disease and that it may recur in certain periods	Cohort	6
39	Farouk et al., 2017 [50]	Egypt	30	31.6	0	15.5	Rifampicin is effective in the treatment of patients with IGLM	Cohort	7
40	Freeman et al., 2017 [51]	USA	14	31.7	—	3	Granulomatous mastitis is uncommon and difficult to diagnose and manage. We review our experience, the literature, and propose an algorithm for diagnosis and management	Cohort	5
41	Shin et al., 2017 [52]	South Korea	34	35	6	37.6	Wide excision resulted high recurrence than steroid and drainage groups and left extensive scarring	Cohort	7
42	Co et al, 2018 [53]	Hong Kong	102	33	12	14	IGM is uncommon with a recurrence rate of 12%, especially in patients with history of smoking and isolation of *C. kroppenstedtii*	Cohort	7
43	E. Uysal et al., 2018 [15]	Turkey	720	36	122	16	Our findings show that history of pregnancy, breastfeeding, breast infection, and smoking were the risk factors for IGM recurrence	Cohort	7
44	Cetin et al., 2019 [54]	Turkey	124	33.9	17	21.9	Topical steroids would be among first-line treatment options of IGM	RCT	4
45	Chen et al., 2019 [55]	China	32	32	3	15.6	Ductal lavage for patients with NLM is feasible and safe, and a definitive randomized controlled trial for further investigation is warranted	RCT	3
46	Davis et al., 2019 [56]	USA	120	35	19	20	Idiopathic granulomatous mastitis is a self-limited, benign condition that waxes and wanes and eventually resolves without resection	Cohort	8
47	Kaviani et al., 2019 [57]	Iran	374	34.6	—	—	The outcome of prednisolone use in severe cases was comparable to NSAIDs	Cohort	8
48	Li, 2019 [58]	China	75	35.9	3	6	Surgery and symptomatic treatment can completely remove the lesions inorder to cure the disease	Cohort	7
49	Yaghan et al., 2019 [59]	Jordan	68	37.75	19	10	Treatment of IGM in any institution should be the responsibility of a multidisciplinary team	Cohort	6
50	Azizi et al., 2020 [6]	Iran	474	33.9	118	—	The recurrence rate was 24.8%, and breast skin lesions were associated with a significantly higher odds of recurrence	Cohort	6
51	Haddad et al., 2020 [60]	Iran	17	36.68	3	16.4	For those patients with IGM who are not candidates for surgical intervention or require corticosteroid‐sparing medical treatment as well as those whose symptoms recur after tapering of their initial treatment, MTX‐based treatment could be an attractive alternative therapeutic option with favorable outcome and less frequent side effects	Cohort	6
52	Kehribar et al., 2020 [61]	Turkey	33	36.9	0	24	Steroid + methotrexate treatment is an effective and reliable method for ensuring long-term remission in patients with idiopathic granulomatous mastitis diagnosis	Cohort	6
53	Montazer et al., 2020 [62]	Iran	30	34.8	3	12	High dose prednisolone has a high success rate and a lower recurrence in the treatment of IGM and could reduce the need for surgery	RCT	4
54	Postolova et al., 2020 [63]	USA	19	33.5	3	36	MTX monotherapy is an effective treatment for IGM	Cohort	7
55	Steuer et al., 2020 [64]	USA	32	35.6	—	12	Adequate medical management may alleviate the need for surgical intervention	Case series	9
56	Tekgöz et al., 2020 [65]	Turkey	53	37.2	5	13.83	Methotrexate seems to be efficient in the treatment of idiopathic granulomatous mastitis and provides drug-free remission	Cohort	7
57	Zhang et al., 2020 [66]	China	53	34.6	4	12.6	Surgical management combined with postoperative oral Yanghe decoction treatment yielded a higher CR rate and lower recurrence rate than surgery alone	Cohort	6
58	Emsen et al., 2021 [67]	Turkey	51	37	—	—	The observed changes in T cells, NK, and NKT cells suggest that there is systemic immune dysregulation in patients with IGM	Case control	7
59	Koksal, 2021 [68]	Turkey	134	33.5	10	—	Clinical differences were detected among the patients with IGM, and classification of patients by severity is needed to plan the optimal treatment approach	Cohort	5
60	Ringsted and Friedman, 2021 [69]	Portland	28	32	7	27	MTX is a promising treatment for IGM	Case series	9
61	Shojaee et al., 2021 [70]	Iran	87	34.11	25	26	The use of minimally invasive methods such as drainage plus low-dose steroids is a more effective method with fewer side effects than the other two methods	Cohort	5
62	Tang et al., 2021 [71]	Singapore	77	36	—	18	Smoking is associated with higher number of flares of IGM and should be discouraged in IGM patients	Cohort	7
63	Velidedeoğlu et al., 2021 [72]	Turkey	86	31.85	11	29.65	All physicians should not neglect questioning breast complaints in patients with EN since EN may be caused by IGM	Case control	8
64	Koksal, 2022 [73]	Turkey	61	36	—	—	Classification of patients by severity is needed to plan the optimal treatment approach	Cohort	6
65	Li et al., 2021 [74]	China	15	30.5	—	—	Observational therapy during pregnancy for PAGM is reliable and feasible	Case series	6
66	Dalbaşı and Akgül, 2021 [75]	Turkey	62	36.5	7	24	Methotrexate + low-dose steroid therapy is successful in the treatment of IGM	Cohort	6
67	Karami et al., 2021 [76]	Iran	118	34	12	10	Local betamethasone LA injection in breast-limited IGM is as successful as current standard treatment and shortens the complete healing time compared to treatment with systemic therapy	RCT	4
68	Bayrak et al., 2021 [77]	Turkey	77	39.24	28	24	A detailed assessment accompanied with clinical, radiological, and pathological findings should be performed to achieve an accurate diagnosis and effective patient management in IGM	Cohort	6
69	Pala et al., 2022 [78]	Turkey	114	35.8	15	—	For optimal management and timing the appropriate therapy, the ideal biopsy procedure, special stains, and a multidisciplinary team consisting of the surgeon, pathologist, and radiologist are the most important issues	Cohort	5
70	Soltany et al., 2022 [79]	Syria	17	37.2	4	—	The histopathological study is considered as the most crucial element in the multidisciplinary approach to diagnose idiopathic granulomatous mastitis (IGM) and determine the optimal management to be administered	Cohort	5
71	Velidedeoğlu et al., 2022 [80]	Turkey	152	—	51	—	IGM is typically seen in women of childbearing age with a recent history of pregnancy and lactation	Cohort	5

Ref: reference, Rec: recurrence, f/u: follow-up. Newcastle–Ottawa, JBI risk of bias, and Jadad checklists were used for the quality assessment of cohort/case-control, case series, and randomized controlled trial (RCT) studies.

**Table 2:** Study characteristics: insights from parameter studies.

Parameter	Studies	*n*	Mean or frequency (percentage)
*Race*			
Caucasian	71	4735	3749 (79.17%)
Others			986 (20.83%)
Age	69	4566	34.98 years
Parity	55	3554	3251 (91.47%)
Lactation	44	3252	2721 (83.67%)
OCP	41	3203	730 (22.79%)
Smoking	38	2687	373 (13.88%)
Size	37	2151	4.64 cm
Trauma	16	628	36 (5.7%)
BMI (mean)	4	198	28.24
*BMI*			
<25	4	146	55 (37.67%)
>25			91 (62.32%)
*BML*			
<30	3	164	76 (46.34%)
>30			88 (63.66%)
Recurrence rate	60	4038	694 (17.18%)

**Table 3:** Comparison of different variables based on the race.

Parameter	Study		Race		*p* value
	Caucasian	Others	Caucasian	Others	
Recurrence	42	18	599/3214 (18.63%)	95/824 (11.52%)	0.171
Parity	36	19	2679/2917 (91.8%)	572/637 (89.79%)	0.621
OCP	30	11	671/2857 (23.48%)	59/346 (17.05%)	0.289
Lactation	31	13	2322/2752 (84.37%)	399/500 (79.80%)	0.462
Mass	30	17	2352/2941 (80%)	595/745 (79.86%)	0.999
Pain/tenderness	24	18	1327/2520 (52.65%)	397/750 (52.93%)	0.998
LAP	20	2	372/1881 (19.77%)	43/108 (39.81%)	0.003
Inflammation	25	20	863/2501 (34.5%)	179/615 (29.1%)	0.396
Skin	28	7	446/2622 (17%)	35/209 (16.74%)	0.999
Abscess/fistula/sinus	36	17	1318/4299 (30.65%)	143/645 (22.17%)	0.380
Nipple changes	23	15	300/2541 (11.8%)	89/576 (15.45%)	0.535
Systemic	7	2	90/1222 (7.36%)	6/60 (10%)	0.434

**Table 4:** Comparison of recurrence rates based on different treatments.

	Type of treatment						*p* value
	All	Observation	AB^*∗*^	Immunosuppressive	Surgical	Combined	
Recurrence	694	5	12	114	125	78	0.023
*n*	4038	53	178	774	555	523	
Rec rate	17.18%	9.4%	6.74%	14.7%	22.5%	14.9%	
*p* value in comparison to surgical		0.013	0.003	0.194		0.190	

^*∗*^Antibiotic.

## Data Availability

All data generated or analyzed during this study are included within the article. Further inquiries can be directed to the corresponding author.
